# Recovery of Nutrients from Cod Processing Waters

**DOI:** 10.3390/md21110558

**Published:** 2023-10-26

**Authors:** Jorge Coque, Charlotte Jacobsen, Bita Forghani, Anders Meyer, Greta Jakobsen, Jens J. Sloth, Ann-Dorit Moltke Sørensen

**Affiliations:** 1 National Food Institute, Technical University of Denmark, 2800 Kongens Lyngby, Denmark; 2 Food and Nutrition Science, Biology and Biological Engineering, Chalmers University of Technology, 41296 Gothenburg, Sweden; bita.forghani@chalmers.se; 3 Jeka Fish A/S, 7620 Lemvig, Denmark; contact@jeka-group.com; 4 Højmarklaboratoriet A/S, 6940 Lem St., Denmark; kontakt@hojmarklab.dk

**Keywords:** protein, phosphorus, flocculation, flocculants, ultrafiltration, microfiltration

## Abstract

Liquid side-streams from food industries can be processed and used in food applications and contribute to reduce the environmental footprint of industries. The goal of this study was to evaluate the effectiveness and applicability of protein and phosphorus separation processes, namely microfiltration, ultrafiltration and flocculation, using protein-rich process waters with low (LS) and high (HS) salt content from the processing of salted cod (Gadus morhua). The application of different flocculants (chitosan lactate and Levasil RD442) were evaluated at different concentrations and maturation periods (0, 1 or 3 h). The results showed that different flocculation treatments resulted in different recoveries of the nutrients from LS and HS. Proteins in LS could be most efficiently recovered by using Levasil RD442 0.25% and no maturation period (51.4%), while phosphorus was most efficiently recovered when using Levasil RD442 1.23% and a maturation period of 1 h (34.7%). For HS, most of its protein was recovered using Levasil RD442 1.23% and a maturation period of 1 h (51.8%), while phosphorus was recovered the most using Levasil 1.23% and no maturation period (47.1%). The salt contents allowed interactions through intermolecular forces with Levasil RD442. The ultrafiltration method was effective on HS since it recovered higher percentages of nutrients in the retentate phase (57% of the protein and 46% of the phosphorus) compared to LS.

## 1. Introduction

The production of marinated, salted fish generates huge amounts of process water, which is one type of liquid side-stream from the food industry. Liquid side-streams in the fish industry are produced from different sources, such as the seawater used for transportation after the capture of fish, water used in the filleting process, frozen fish thawing, etc. Each of them is produced in different quantities and has its own profile of nutrients. The volume of liquid side-stream (wastewater) generated by seafood processing operation varies depending of the process from <1 up to 44 m^3^ per ton of fish raw material [1]. For the processing of brined herring, the brine can count for more than 1000 m^3^ of wastewater per ton of produced fish [2]. For years, brines and other process waters have been discarded without any attempt to recycle it or recover nutrients from it; and companies have faced the high costs of mandatory treatment due to environmental regulations [2]. Thus, the seafood processing sector contributes serious organic pollution loads with these amounts of wastewater generated [1]. The process waters are food grade and follow hygienic standards, making it realistic to recover molecules of interest [2]. This would lead to an important reduction in environmental pollution and additional revenue for the producing company. The risk of eutrophication in natural water sources into which these side-streams are discharged could also be reduced if phosphate is successfully recovered. Furthermore, the recovery of protein means that the chemical oxygen demand (COD) might be largely reduced in the wastewater treatment [3].

Several techniques have been applied to recover nutrients from process water such as flotation, flocculation, chemical precipitation and membrane-based separation processes [4,5,6]. Cleaning of the process water by using it as growth media (nutrients) for algae cultivation has also been attempted [4]. Moreover, membrane technologies, such as ultrafiltration or the use of flocculants are two other methods that can be used to recover proteins [7]. Several studies have described the recovery of phosphate for conversion into the fertilizer struvite [8] for conversion into a form that can be directly used by plants [9]. There is a need to recover phosphate in its pure form, and membrane technology is an option [10].

Coagulation/flocculation is the liquid–solid separation process most widely used to remove dissolved and suspended solids, organic matter and colloids present in industrial process waters [11]. It uses the salts of cationic inorganic metals as coagulants, while anionic or non-anionic polymers are used as flocculants [12]. It has been used in sanitary landfill leachates, pulp mill wastewater, palm oil mill effluent, oily wastewater, textile wastewater and others [13]. On the other hand, direct flocculation uses a medium charge density with cationic polymers of high molecular weight as flocculants, and coagulants are not needed [12]. Both consist of the aggregation or agglomeration of dispersed or finely divided particles to form larger particles (flocs) able to settle, clarifying the system [14], and involve charge neutralization [13], polymer bridging [15] and formation of “islands” or “patches” [16] as molecular mechanisms. Flocculation has earlier been studied in process waters for recovery of protein and lipids, where flocculation with carrageenan or alginate improved the lipid and protein recovery in combination with dissolved air flotation (DAF) compared to only DAF or DAF in combination with acid precipitation [5,6].

The processes that involve membrane technology in the treatment of wastewaters are an alternative to the traditional methods such as sedimentation, oxidation and anaerobic treatments. In particular, the application of membrane bioreactors (MBRs) for treating wastewater has received increasing attention [17]. The membranes are devices with different compositions and pore sizes, which separate solids from a liquid stream. Microfiltration (MF) with pore sizes of 0.1–10 µm and ultrafiltration (UF) with pore sizes of 0.01–0.1 µm are the most commonly used membrane technology for the treatment of wastewater in the food industry [18]. Microfiltration is used to remove bacteria, suspended solids and large molecules without having to use any chemicals; this is why it is preferable to treat pre-treated and raw food processing wastewater, even though protein can pass the membrane [10,19]. It does not chemically modify pollutants, but its main role is to fractionate the components of wastewater and turn it into a less polluting and more useful stream [20]. Ultrafiltration is nowadays most commonly used in biotechnology for the treatment of drinking water and in the dairy industry to obtain concentrates of whey protein from the processing of cheese [21,22]. In membrane processes, the membrane is always in contact with the solutions being treated, which consequently can cause deposition and accumulation of materials on or in the membrane also referred to as membrane fouling. There are four types of fouling: (1) complete pore blocking, (2) partial pore blocking, (3) internal pore blocking and (4) cake formation. Membrane fouling will cause a decline in flux, due to material accumulated on the membrane surface or inside the membrane. Thus, the membrane performance is degraded [4,23]. Several studies have shown that this technology can be applicable for recovery of nutrients from process water [24,25].

Salted cod is an important product category in, e.g., Southern Europe, where it is known as Bacalao. In the production of salted cod, liquid side-streams are generated several places in the process line with different contents of nutrient. In Denmark, the production of salted cod yearly generates around 15,650 m^3^ of process water (liquid side-stream/wastewater rich in nutrients). The aim of this work was to compare and evaluate the effectiveness and applicability of different separation processes to recover protein and phosphate from process water generated during production of salted cod (Bacalao), namely microfiltration, ultrafiltration and flocculation with different concentrations of flocculant (chitosan lactate and Levasil RD442) and different maturation periods. The different process waters were selected primarily on the basis of their salt and protein contents in order to evaluate the effect of the salt concentration on the amount of recovered nutrients. Overall, we evaluated the hypothesis that due to a smaller pore size, ultrafiltration will more effectively separate proteins and phosphate from the process waters and concentrate them in the retentate, i.e., higher recovery, than microfiltration. For the flocculation technology, the aim was to evaluate the hypothesis that higher concentrations of flocculants and longer maturation periods will sediment more protein and phosphate than lower concentrations and shorter maturation time, since more particles of flocculants will be able to interact with most of the molecules present in the process waters during more time to form flocs and settle.

## 2. Results and Discussion

### 2.1. Side-Stream Characterization

At Jeka Fish (Lemvig, Denmark), several streams of process waters are available. Side-stream waters are process waters obtained from different stages in the process of producing salted cod. The company uses approximately 16,500 tons of water per year in different process steps, which can contain around 102 tons of protein in total. Two different process waters with high protein content and either low salt content (LS) or high salt content (HS) were selected for this study (Table 1). Sample LS is the process water collected on the same day the fish was initially put in the salting solution, while sample HS belongs to the process water that was dripping from the cod during the entire salting period. Table 2 describes the protein content of the process waters that were used for the separation processes in this study. The results show that for the LS process water, protein contents varied substantially (0.11 to 0.41%), whereas protein contents in HS were more stable (0.28% to 0.38%). Differences in protein percentages of LS and HS between Table 1 and Table 2 could be explained by the different method used to determine protein content, since the information presented in Table 1 was based on results after using the Kjeldahl method, while the information presented in Table 2 was based on results after using the Bicinchoninic Acid (BCA) method, which only measure the soluble proteins.

### 2.2. Flocculation Treatments

#### 2.2.1. Protein Sedimentation

**Figure 1:**
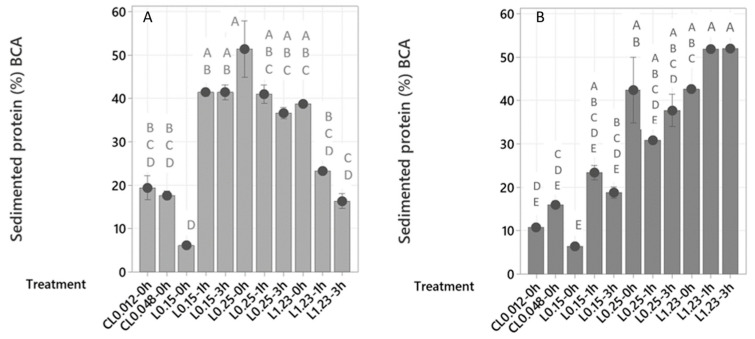
Protein recovery in the sediment (%) calculated from protein content using the BCA method, after treatments with chitosan lactate 0.012% and no maturation period (CL 0.012–0 h), chitosan lactate 0.048% and no maturation period (CL 0.048–0 h), Levasil RD442 0.15% with 0, 1 and 3 h of maturation period (L0.15–0 h, L0.15–1 h, L0.15–3 h), Levasil RD442 0.25% with 0, 1 and 3 h of maturation period (L0.25–0 h, L0.25–1 h, L0.25–3 h) and Levasil RD442 1.23% with 0, 1 and 3 h of maturation period (L1.23–0 h, L1.23–1 h, L1.23–3 h) of process waters LS (**A**) and HS (**B**). Values of sedimented protein (%) or protein recovery in sediment (%) are shown as means, and error bars indicate the standard deviations (*n* = 2 and *n* = 4 for L0.25–0 h for both LS and HS). The capital letters above the bars are the results from the Tukey analysis and different letters indicate significant differences.

The protein recovery in the sediment (%) obtained after different treatments are shown as the means of sedimented protein (%) for each treatment in Figure 1. Levasil RD442 was more successful than chitosan lactate in recovering protein in the sediment in both process waters. This is explained by the fact that chitosan lactate becomes a cationic polymer that is soluble and with a high charge density when the pH of the solution is below 6 [26]. The pH of the samples used for this experiment was 7.2 in LS and 6.1 in HS. Thus, the pH values above 6 in the samples did not allow an electrostatic interaction between the chitosan chains and the charged proteins in the process waters. Treatments with chitosan lactate might need a pH adjustment step, which was not performed. On the other hand, the treatment of LS with Levasil RD442 0.25% without a maturation period resulted in the highest value of protein recovery in the sediment or sedimented protein (51.4%), being significantly higher than the results obtained after the treatments with chitosan lactate and with Levasil RD442 1.23% and maturation periods of 1 and 3 h. The treatment of LS with Levasil RD442 0.15% without a maturation period resulted in the lowest value of sedimented protein (6.1%), being significantly lower than the results obtained after the treatments with Levasil RD442 0.15% and maturation periods of 1 and 3 h, with all the treatments with Levasil RD442 0.25%, and with Levasil RD 1.23% without a maturation period. In HS, the flocculation with Levasil RD442 1.23% and maturation periods of 1 and 3 h allowed the highest values of sedimented protein (51.8% and 51.9%, respectively), being significantly higher than the results obtained after the treatments with chitosan lactate and with Levasil RD442 0.15% with 0 and 3 h of maturation period. Similarly to LS, the treatment with Levasil RD442 0.15% of HS without a maturation period resulted in the lowest value of sedimented protein (6.4%), being significantly lower than the results obtained after the treatments with Levasil RD442 0.25% and maturation periods of 0 and 3 h, and after all the treatments with Levasil RD442 1.23%. A comparison of treatments that recovered most protein in process waters LS and HS showed that both resulted in a recovery of ca. 51% of the protein in the sediment.

**Figure 2:**
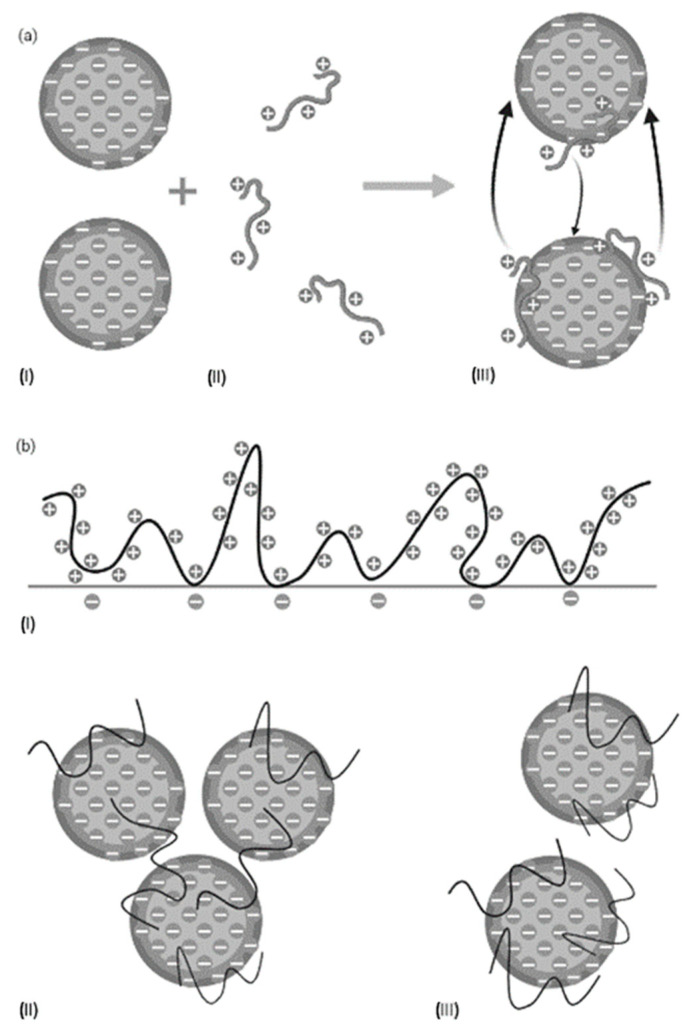
Flocculation mechanisms. (**a**) Electrostatic patch mechanism. (**I**) Particle with negative charge. (**II**) Flocculants with high charge density and low molecular weight. (**III**) Attraction of opposite charges. (**b**) Polymer bridging mechanism. (**I**) Polymer adsorption and loop formation. (**II**) Polymer bridging with other particles. (**III**) Restabilization. (Modified from Sharma et al., 2006 [19]).

The protein recovery in the sediment (%) showed that the most effective sedimentation after flocculation with Levasil RD442 depended on many factors, because the tendencies were not the same for the different process waters, LS and HS. This is attributed to the characteristics of the flocculant, including the protein and salt content of the process waters, time of maturation and intermolecular forces that were formed during the flocculation process. As a reference, it is known that the fish protein net charge is at its isoelectric point when the pH of their solution is 5.0–6.0, and this value can shift towards the acidic range when the fish proteins are in salty waters, depending on the salt concentration [27]. A protein has a positive charge when the pH of the solution is below the isoelectric point, while it has a negative charge when the pH of the solution is above the isoelectric point [16]. The process waters used in the experiments had pH values above 6.0; therefore, the proteins in the process waters had a negative charge. This can be used to explain the interactions between the proteins, and the substances involved in the treatments (salts and flocculants). The results of treatments with Levasil RD442 (Figure 1) showed that higher concentrations of flocculant did not mean that there was a higher protein sedimentation for LS. This fact may be explained by the flocculation mechanism that the spherical nanoparticles of amorphous silica contained in Levasil RD442 are proposed to follow. These nanoparticles have high surface electrical conductivity and low molecular weight [14], suggesting that the flocculation mechanism was through electrostatic patch [16], and if the flocculant concentration increases it would be through polymer bridging because they would act like long chain polymers [15]. As shown in Figure 2a, the flocculants surround the surface of the particles in the process waters. It is possible that higher concentrations of flocculants overcoated the protein surfaces for LS (Figure 2b) not allowing the dipole–dipole intermolecular force [28] to be formed between flocculants and neighbor proteins, which is translated into less sedimentation of the protein.

It is also clear that process water LS sedimented more protein than HS when there was no maturation period, which could be due to the salt content of the process waters. As shown in Table 1, LS had a salt content between 6.95% and 13.44%, while the salt content in HS was between 22.75% and 25.63%. Salts also have electric charges and their presence would bring electrical interactions through intermolecular forces (ion–dipole) [28] with the flocculants, which would interfere in the interaction between flocculants and protein that can mostly exist in samples with lower salt content. When the maturation period increased in the flocculation treatments, the behavior was different. In process water HS, the electrostatic attraction between the salts and Levasil RD442 mostly existed at the beginning, but the negatively charged proteins provided a stronger attraction with the flocculant (dipole–dipole), and the maturation period was necessary for its formation, where 1 h was the optimal maturation time for HS. The interaction between salts and the flocculant (ion–dipole) seemed to prevent overcoating of the protein when higher flocculant concentrations were applied. However, it is possible that a higher concentration of Levasil RD442 reduces the protein sedimentation if the overcoating cannot be avoided, since the protein sedimentation did not increase from 2 to 3 h of maturation time. In the case of process water LS, where there was much lower salt content, the electrostatic interaction between the proteins and the flocculant decreased with longer maturation time and higher concentrations of flocculant. The chance of the flocculant to overcoat the protein increases if there is less possibility of interaction between salts and flocculant particles in excess; therefore, the interactions (dipole–dipole) results in lower formation of protein flocs.

Summarizing, lower salt content in the process water led to an interaction only between the proteins and flocculant, where excess of flocculant and maturation time negatively affected the sedimentation of protein due to increased chance to overcoat the protein. This is why the optimal treatment for sample LS was with Levasil RD442 0.25% and no maturation period. In contrast, higher salt content led to interactions between proteins and flocculant, but also to complementary electrostatic interactions between the salts in the process water and the flocculant. A higher concentration of flocculant and maturation time positively affected the sedimentation of protein up to a certain point due to controlled overcoating of the protein. This is because of the presence of interactions between salts and excess of flocculant. Thus, the optimal treatment for HS was with Levasil RD442 1.23% and 1 h of maturation period.

Wibowo et al., 2005 [29] determined that a concentration of 100 mg/L of the chitosan-alginate complex for 1 h achieved a protein recovery of 83% from surimi wash water. Forghani et al., 2020 [6], reduced the protein concentration of shrimp boiling water (SBW) by up to 76%, while shrimp peeling water (SPW) resulted in a protein reduction by up to 85%, using alginate and carrageenan as flocculants combined with dissolved air flotation (DAF). The biomass collected after the process of SBW and SPW had 7 and 29 times more protein, respectively, than their initial waters. Similarly to our findings for the LS and HS process water, the results from the boiling and peeling water also showed that the recovery depended on the type of process water. In addition, flocculation with carrageenan combined with DAF resulted in higher protein and lipid recovery than without flocculation or with only acidification [5]. Pereira et al., 2022 [7], discovered that the use of carrageenan as flocculant at a concentration of 60 mg/L and chitosan as coagulant at a concentration of 600 mg/L recovered 71% of the protein and 82% of lipids from sardine cooking waters. The values of protein recovery of these studies are higher than the protein recovery reached by the use of Levasil RD442 in the presented work, since the most successful treatment recovered no more than 52% of the protein despite being similar to lower concentration of flocculant applied in the former studies.

#### 2.2.2. Polypeptide Profiling Using Sodium Dodecyl Sulfate—Polyacrylamide Gel Electrophoresis (SDS-PAGE)

**Figure 3:**
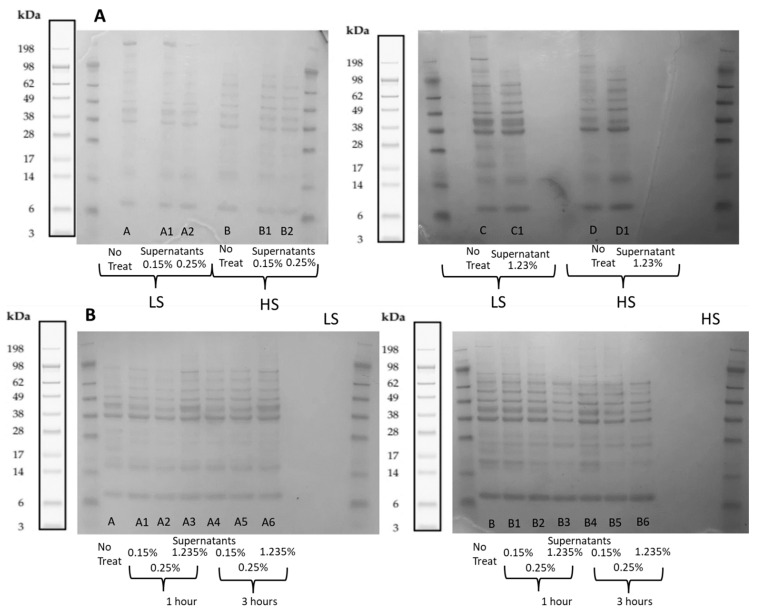
Polypeptide profiling before and after the treatment (supernatants) of process waters LS and HS with Levasil RD442. (**A**) Without maturation period. Sample abbreviation: A and C refer to no treatments of LS, and A1, A2 and C1 refer to supernatants of 0.15, 0.25 and 1.23% Levasil treatment of LS, respectively; B and D refer to no treatments of HS, and B1, B2 and D1 refer to supernatants of 0.15, 0.25 and 1.23% Levasil treatment of HS. (**B**) With maturation periods of 1 and 3 h. Sample abbreviations: A refers to no treatment of LS, and A1, A2 and A3 refer to supernatants of 0.15, 0.25 and 1.23% Levasil treatments of LS with 1 h maturation, and A4, A5 and A6 refer to supernatants of 0.15, 0.25 and 1.23% Levasil treatments of LS with 3 h maturation; B refers to no treatment of HS, and B1, B2 and B3 refer to supernatants of 0.15, 0.25 and 1.23% Levasil treatments of HS with 1 h maturation, and B4, B5 and B6 refer to supernatants of 0.15, 0.25 and 1.23% Levasil treatments of HS with 3 h maturation. The polypeptide profiles are compared with a high-molecular weight (HMW) standard. An image of bands from the manufacturer of the protein mix of the standard is included on the left and right of each gel.

Polypeptide profiling was performed using SDS-PAGE, as shown in Figure 3. An explanation for the presence of weaker bands after the treatments is given by the fact that this analysis was performed on the supernatants obtained after the flocculation treatments, which confirmed that the proteins were sedimented and present in the precipitate phase, which was not analyzed. Those bands that completely disappeared describe the polypeptides that were mostly sedimented and the most effective treatments to sediment proteins, which are supported by a lower protein amount in the supernatant measured via the BCA method.

Polypeptides in the process waters, LS and HS, before and after the treatments with Levasil RD442 at different concentrations without a maturation period are shown in Figure 3A. It was possible to identify that process waters LS and HS without treatment had some differences in their molecular weight distribution of the polypeptides present. The gel showed a clear band above 198 kDa for LS; this one could not be observed in HS. Also, process water HS showed more bands between 38 and 98 kDa than LS.

The treatment with Levasil RD442 0.15% in LS did not alter the molecular weight distribution, since the bands remained after the treatment. But there were changes in the molecular weight distribution of polypeptides when the concentration of flocculant increased. When Levasil RD442 was at 0.25%, the band at 198 kDa slightly faded, while it completely disappeared when the concentration of flocculant was 1.23%. Moreover, some bands between 14 and 38 kDa became weaker. It makes sense that the general intensity of the bands was the weakest with a Levasil concentration of 0.25%, since this was the treatment that was more effective to sediment protein according to the protein content measured with the BCA method. The case of process water HS was the opposite to LS, since the increase in concentration of the flocculant did not alter the molecular weight distribution. Thus, the same bands could be identified in treated and untreated samples, although bands were slightly less intense in treated samples.

The molecular weight distribution of polypeptides in process waters LS and HS with the same previous concentrations of Levasil RD442 (0.15%, 0.25% and 1.23%), but with maturations periods of 1 and 3 h, as shown in Figure 3B. In this case, the observed difference in the previous experiment between the untreated process waters was the opposite to when there was no maturation period, since the band above 198 kDa could be seen in process water HS and not in LS. The intensity of the bands of LS slightly decreased when the concentration of Levasil RD442 was increased, especially with a concentration of 0.25% and maturation period of 1 h. Also, the bands from process water with a maturation period of 3 h seemed more intense than the untreated process water. There was a band with weak intensity between 17 and 28 kDa in the untreated process water LS that could not be seen after all the treatments. The case of process water HS was more uniform in the fact that the increased concentration of Levasil RD442 and longer maturation periods resulted in fading of the intensity of the bands. This could be mostly recognized in the treatments with Levasil RD442 1.23% with a maturation period of 1 h and 3 h compared to the untreated sample, which showed the weakest intensity of bands. In these last treatments it was also observed that band above 198 kDa completely disappeared. Since it is a bigger molecule, it tended to form flocs more easily due to higher surface electrical conductivity that would allow more interactions with the flocculants, and therefore, this large protein was completely sedimented and thus not present in the supernatant.

#### 2.2.3. Phosphorus and Other Minerals Sedimentation

**Figure 4:**
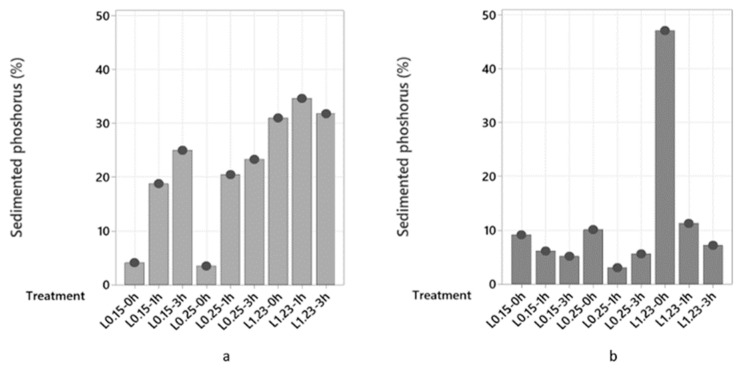
Sedimented phosphorus (%) after treatments with Levasil RD442 0.15% with 0, 1 and 3 h of maturation period (L0.15–0 h, L0.15–1 h, L0.15–3 h), Levasil RD442 0.25% with 0, 1 and 3 h of maturation period (L0.25–0 h, L0.25–1 h, L0.25–3 h) and Levasil RD442 1.23% with 0, 1 and 3 h of maturation period (L1.23–0 h, L1.23–1 h, L1.23–3 h) of process waters LS (**a**) and HS (**b**).

**Figure 5:**
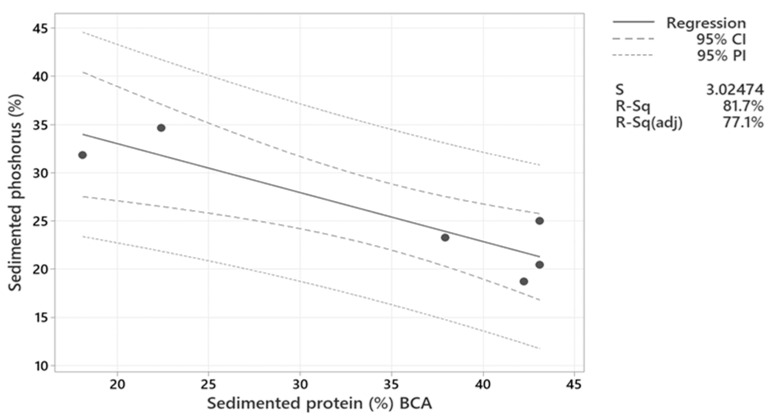
Linear regression between sedimented phosphorus (%) and sedimented protein (%) after treatment with Levasil RD442 at concentration of 0.15%, 0.25% and 1.23% and maturation periods of 1 and 3 h of process water LS. Sedimented phosphorus (%) = 43.15–0.5071 × (Sedimented protein (%) BCA). The variables have a statistical difference (*p* = 0.013), and there is a strong relationship between them (R-Sq = of 81.7%) and a negative slope, which means that the sedimentation of phosphorus depend on the protein sedimentation, being inversely proportional.

Because chitosan could not efficiently sediment proteins, its ability to sediment phosphorus was not investigated. Figure 4 shows the phosphorus sedimentation in process waters LS and HS treated with different concentrations of Levasil RD442 and maturation periods. Process water HS sedimented more phosphorus than LS when there was no maturation period, but the opposite occurred with maturation periods of 1 and 3 h. For process water LS, there was a clear tendency to increase sedimentation when the concentration of Levasil RD442 and time of maturation period increased (Figure 4a). This fact corresponded to the decrease in protein sedimentation, since again overcoating of the protein allowed effective interaction of the few salts present in LS. This relationship could be confirmed with the linear regression with negative slope and high regression coefficient (R-Sq = 81.7%) presented in Figure 5. On the other hand, phosphorus sedimentation slightly increased for HS when the flocculant concentration was increased in samples, but the increase in maturation period decreased the phosphorus sedimentation (Figure 4b). This lack of correlation (R-Sq = 45.1%) could be due to the presence of other minerals, such as Mn, Mg and Na, among others, that also had interaction with Levasil RD442 (Table S1).

The treatment with Levasil RD442 at a concentration of 1.23% and maturation period of 1 h resulted in the most efficient sedimentation of phosphorus (34.7%) in process water LS. In the case of process water HS, treatment with Levasil RD442 at a concentration of 1.23% without a maturation period sedimented most phosphorus (47.1%), and even more than in LS.

Phosphorus in the form of phosphate behaves as a salt. Thus, its recovery followed the principles addressed in Section 2.2.1. And this explains the behavior of the phosphorus sedimentation observed (Figure 4). Even though there were other salts in the process waters, the same phenomena that governed the protein recovered also matched the findings for the phosphorus as a salt (phosphate). Figure 4 shows an increase in the sedimentation of phosphorus when the concentration of Levasil RD442 was increased from 0.25% to 1.23%, both without a maturation period. This corresponded to the decrease in protein sedimentation in both process waters due to the protein overcoating, in which salts took advantage to form interactions with the excess flocculant. Also, the sedimentation of phosphorus was higher in HS than in LS, while the protein sedimentation was higher in LS. An explanation is given by the higher salt content in HS, which when including the phosphates as a salt, could form more interactions with the flocculant and settle.

Sedimentations of the other minerals (Na, Mg, P, K, Ca, Mn, Fe, Cu, Zn and Cd) using Levasil RD442 as flocculant were also investigated (Table S1). A sedimentation of more than 50% was reached for Mg in both process waters, as well as for Mn and Zn in process water LS, and Cu in process water HS. The rest of the minerals (Na, K and Fe) reached lower sedimentations in both process waters (Table S1). For LS, the treatments with Levasil RD442 0.15% and 0.25% and maturation periods of 1 and 3 h did not recover Na, while treatments with Levasil RD442 1.23% and maturation periods of 1 and 3 h recovered 4.7% and 4.5%, respectively. For HS, the treatments with Levasil RD442 0.15%, 0.25% and 1.23% with a maturation period of 1 h recovered 3.8%, 3.9% and 5.8% Na, respectively, while an increasing maturation period (3 h) recovered 4.1%, 5.3% and 3.2% Na, respectively. Cd was under the limit of quantification.

### 2.3. Membrane Technology Treatments

#### 2.3.1. Protein Recovery

**Figure 6:**
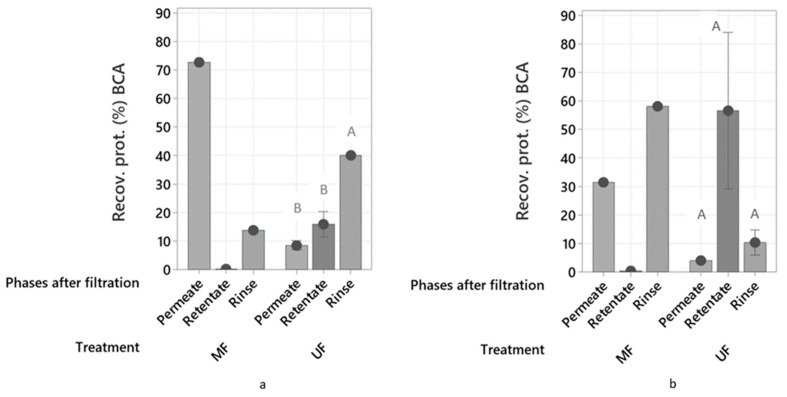
Recovered protein (%) in the phases after filtration calculated from protein content using the BCA method after treatment of process water LS (**a**) and HS (**b**) using microfiltration (MF) and ultrafiltration (UF) membranes. Standard deviations and Tukey analysis are also presented. The capital letters above the bars are the results from the Tukey comparison analysis and different letters indicate significant differences.

The results of the recovered protein (%) in the phases after the ultrafiltration and microfiltration treatments of process waters are presented in Figure 6. After the ultrafiltration processes of LS and HS, the rinse phase of LS had the highest recovery of protein (40.1%), while the retentate phase of HS had the highest recovery of protein (56.6%). In microfiltration treatment, LS recovered most of its protein in the permeate (72.8%), while HS had highest protein recovery in the rinse phase (58.1%). The high recovery of protein in the rinse phase could indicate that fouling had occurred resulting in accumulation of nutrients in the membrane [4,23].

In general, the microfiltration process was not optimal for protein separation. The process water together with its protein content went to the permeate side and no recovery in a separate phase could be made. Also, passing the process water through this membrane (pore size 0.1–10 µm) in this specific device decreased the total protein content since it clearly was retained in the system. On the other hand, the ultrafiltration (pore size 0.01–0.1 µm) was shown to be more efficient for process water HS. The protein stayed in the retentate and could be upconcentrated, and the water went to the permeate side, while less protein was retained in the system. There could be a possible interaction between the particles in process water HS and the protein molecules that makes it denser, which would explain the almost exclusive movement of the proteins to the retentate side, whereby retainment in the system could be avoided, but further studies are required to confirm this. Process water LS also retained more protein in the retentate after the ultrafiltration process than after the microfiltration process, but not as much as HS, and a lot more was retained in the system. Thus, ultrafiltration was less optimal for the recovery of protein from LS.

The fact that it was possible to find protein in the permeate side after the treatment of both samples with an ultrafiltration membrane could be explained by the shape of proteins contained in the process waters. Globular proteins are retained in the membrane while thin, long flexible proteins can pass [30]. A characterization of the proteins in the process waters, describing their structure, would provide a better explanation.

Mameri et al., 1996 [31], found that two ultrafiltration modules could concentrate the protein from 5 to 35 g dm^−3^, decreasing the BOD_5_ by 80%. Benhabiles et al., 2013 [32], showed that the combination of NaOH treatment and ultrafiltration concentrated protein in the retentate, and reduced the chemical oxygen demand (COD) of the permeate by 87%. The combination of electroflocculation and ultrafiltration on four types of herring industry processing waters [33] resulted in a recovery of up to 80% proteins and reduced COD by 70%. The values of protein recovery of these studies are higher than the protein recovery reached by the use of the ultrafiltration membrane in the presented work, since the most successful treatment recovered no more than 60% of protein in the retentate.

The loss of protein in the system that was confirmed after finding protein in the rinsing steps resulted in the conclusion that the lab device used to perform the ultrafiltration was not optimal. Also, a better understanding on the pressures to use during the filtration would provide a better outcome. It is possible that the different samples of process waters have different pressure requirements for optimized protein recovery, and therefore, further studies are necessary. Finally, closing the retentate valve completely during the filtration (dead-end filtration), as applied in this work, may not be the best option, because it led to clogging (fouling) of the membrane. Future studies may consider to partially open this valve and repeat the filtration several times.

#### 2.3.2. Polypeptide Profiling Using SDS-PAGE

**Figure 7:**
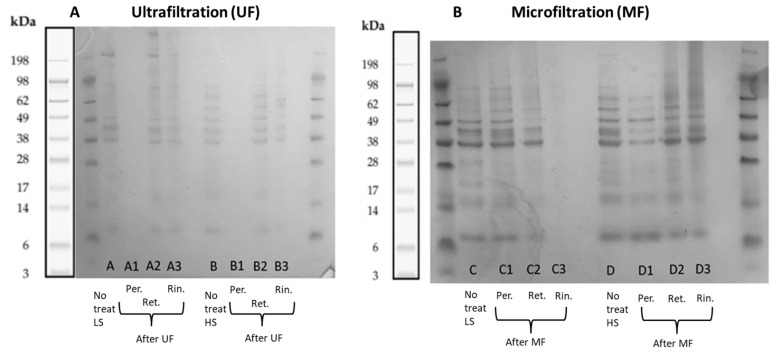
Polypeptide profiling before and after treatment of process waters LS and HS with membrane technology compared with a high-molecular weight (HMW) standard. (**A**) Ultrafiltration (UF) and (**B**) microfiltration (MF). After the treatments, the permeate phase (Per.), the retentate phase (Ret.) and a sample from the rinse step (Rin.) were analyzed. An image of bands from the manufacturer of the protein mix of the standard is included on the left of each gel.

Figure 7 shows the molecular weight distribution of polypeptides in LS and HS before treatment and in the phases after membrane filtration. It was not possible to detect/visualize bands in the permeate phase after the ultrafiltration process of the two process waters even though still some proteins remained in the permeates according to protein quantification data (Figure 6). This, however, could be due to low protein concentrations for both samples (<0.40 mg/mL). According to the SDS-PAGE methodology, a protein concentration of 1 mg/mL is required for the bands to appear in the gel.

In the case of process water LS after ultrafiltration, the retentate phase showed a clear band above 198 kDa, while the retentate phase after the ultrafiltration process of HS showed a weak band above 98 kDa, and both were not seen for their respective untreated samples. Bands could be identified in the rinse phases after filtration of both process waters, which confirms that there was loss of protein in the system (fouling), but it was observable that more polypeptides with different molecular weights stayed in the rinse phase after the treatment of process water LS, while process water HS did not get retained as much.

On the other hand, bands in the permeate phase were clearly identified after the microfiltration process of both process waters, where it is required to ideally not find proteins. In the case of the process water LS, the permeate phase showed almost the same bands compared to the untreated sample, but a difference was that some bands between 49 and 98 kDa appeared with more intensity. The opposite occurred in the permeate phase after the treatment of HS, in which the bands were weaker. The retentate phase of both samples were identified, but both with less intensity in some bands compared to the untreated process waters. And even though the size of the pores of the microfiltration membrane is large enough to let the proteins pass to the permeate side, it was possible to find polypeptides in the rinse phase, meaning that the device utilized to perform this treatment was not the most adequate. The bands in the rinse phase of process water LS were almost not visible, while it was possible to identify some in the rinse phase of process water HS.

#### 2.3.3. Phosphorus and Other Minerals Determination

In terms of phosphate recovery, it was more effective to perform ultrafiltration in process water HS, since most of it went to the retentate side. This fact, together with a higher recovery of protein in the retentate side after the treatment of HS, made the treatment ideal for HS and not for LS. The goal is to recover phosphate and protein from the process water, and this cannot be performed in the permeate side because great part of the volume is there and there would not be a real separation.

**Figure 8:**
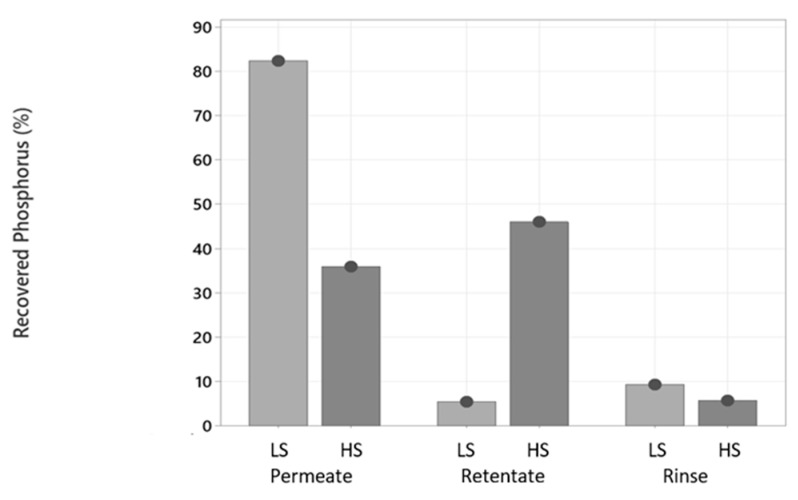
Recovered phosphorus (%) in the phases after filtration of process waters LS and HS from using an ultrafiltration membrane. LS and HS process waters refer to Table 1.

Figure 8 shows that a great part of the phosphorus (82.4%) passed the ultrafiltration membrane in the treatment of process water LS. This made sense because phosphorus in the form of phosphate is an ion that has a size that lets it pass to the permeate side [18] together with other small molecules, such as water. In process water HS, the recovered phosphorus was slightly different in the permeate and retentate phases (36.0% and 46.0%, respectively). As mentioned in the previous section, the higher density with the presence of bigger particles in HS could form intermolecular forces with proteins, that also attached phosphate ions through ion–dipole interaction and brought them to the retentate side, while some were able to pass the ultrafiltration membrane. It is also possible that higher amounts of salts enhanced the interaction between phosphate groups and protein, making them go together, when phosphate was supposed to go directly to the permeate side, as occurred after the ultrafiltration treatment of LS.

Other minerals were measured in the permeate and retentate phases after the ultrafiltration of both process waters. In LS and HS, the concentrations of Na were almost the same in the two different phases after the filtration, as in the untreated water (ca. 30 g/kg in LS and ca. 100 g/kg in HS), showing almost no recovery of Na. Mg, Mn, Fe, Cu and Zn were concentrated in the retentate, mostly Cu and Zn. The value of Cd was below the limit of quantification.

### 2.4. Combination of Technologies

The current study evaluated two different techniques each with different parameters. However, the combination of techniques for an improved recovery also must be evaluated in future. A recent review on macronutrients recovery highlighted that no single technique can effectively and universally recover target macronutrients from liquid waste [4]. From the obtained results, it is also clear that the recovery depends on the composition of the process water treated, i.e., in this study, especially the content of salt. For more effective recovery of nutrients, a future prospective could therefore be a combination of ultrafiltration followed by flocculation. It is likely that it will be possible to lower the concentration of flocculants when the techniques are combined. Other studies of recovery of nutrients from process waters or aquaculture have shown positive results with combination of techniques such as coupling of nanofiltration with osmotic evaporation for recovery of natural flavoring concentrate from shrimp cooking juice [34], flocculation combined with dissolved air flotation [5,6].

## 3. Materials and Methods

### 3.1. Chemicals

The flocculants applied, chitosan lactate and Levasil RD442 (15.22% SiO_2_ solution), were provided by Swedish Hydro Solutions (Alingsås, Sweden) and Nouryon (Amsterdam, The Netherlands), respectively. The BCA kit for protein determination was purchased from ThermoFisher Scientific (Waltham, MA, USA). The gel electrophoresis used a high-molecular weight standard (SeeBlue^TM^ Plus2 Prestained Standard, Thermo Fisher, Whaltham, MA, USA), a running buffer (MES novex NP 0002, Thermo Fisher, Whaltham, MA, USA), a stain reagent (GelCode^TM^ Blue Stain Reagent, Thermo Fisher, Whaltham, MA, USA), Laemlli buffer and dithiothreitol (DDT).

### 3.2. Side-Stream Waters

Jeka Fish (Lemvig, Denmark) collected process waters from their processing facilities (February, March and April 2022). Salt and protein contents of process waters released from each of the steps of the production of salted cod were determined. Process waters LS and HS were selected, as previously mentioned in Section 2. The samples were collected in plastic containers and shipped the same day (cold storage) to the National Food Institute at the Technical University of Denmark. After reception, different containers of the same type of process waters were pooled in one plastic container for each type of process water and stored at 4 °C (≤2 weeks) until further procedures were performed. Previously, it was determined through a total volatile nitrogen (TVN) analysis and microbial count that the process waters can be stored up to 2 weeks without having a risk of oxidation or microbial deterioration (unpublished results). Therefore, the experiments for the samples were performed within this range of time.

### 3.3. Separation of Protein and Phosphorus

#### 3.3.1. Flocculation without Maturation Period

The flocculation was performed according to Forghani et al. (2020) [35] with some modification such as the use of other types of flocculants and no maturation. An amount of 100 mL of 1% chitosan lactate solution was prepared. Then, together with the process waters, 100 mL solutions with final concentrations of 0.012% and 0.048% were prepared. Also, 100 mL solutions with final concentrations of 0.15%, 0.25% and 1.23% of Levasil RD442 based on the concentration of SiO_2_ (15.22%) were prepared with the process waters. These solutions were prepared at cold temperature below 7 °C with stirring (at 200 RPM for 15 min followed by 100 RPM for 60 min). Next, they were centrifuged (1580× *g*, 5 min, 4 °C). After centrifugation, the supernatant was separated from the precipitate, their volumes and masses measured, and the supernatants were transferred to plastic tubes and stored frozen at −40 °C until further analysis.

#### 3.3.2. Flocculation with Maturation Period

The flocculation was performed according to Forghani et al. (2020) [35] with some modification such as the use of another type of flocculant and time of the maturation period. A maturation period was applied for one of the experiments with Levasil RD442 as flocculant. The process waters with Levasil RD442 were stirred at 200 RPM for 15 min followed by 100 RPM for 10 min at temperature below 7 °C. A group of solutions was kept standing for 1 h, while a second group of solutions was kept for 3 h without stirring, i.e., maturation period of 1 and 3 h. After this time, they were centrifuged and treated as mentioned in Section 3.3.1.

#### 3.3.3. Membrane Technology

The Vibro^TM^-Lab3500 device (SANI Membranes, Farum, Denmark) was assembled with microfiltration or ultrafiltration membranes. An amount of 2 L of sample was added to the feed tank, the feed pressure regulator was initially set to 0.4 bar and both the vibration motor and recirculation system pump were turned on. The valve of the retentate outlet was kept closed (dead-end filtration), while the valve of the permeate outlet was kept open for approximately four hours for ultrafiltration and 15 min for microfiltration. It was necessary to adjust the pressure to make the filtration faster or to prevent the membranes from clogging. When there was no more permeate left in the system to be collected, the valve of the retentate outlet was opened and its content was collected in a separate container to be analyzed. To rinse the system, 2 L of distilled water was added to the feed tank. The system was closed and turned on for 5 min, and then both valves of retentate and permeate were opened to collect the rinsing water for later analysis. As a maintenance procedure, after each filtration it was necessary to add to the feed tank 1 L of a 20% solution of ethanol and distilled water, turn the device on for 5 min and leave it there until next use. This last solution was also collected after 24 h. The volumes and masses of the liquids belonging to permeate, retentate, rinsing and ethanol solution that were collected after the treatment were measured, and a fraction of it was put in plastic tubes and stored at −40 °C until further analysis. The nutrients measured from the rinse and maintenance procedures were added and considered as belonging to the rinse phase after the filtrations.

### 3.4. Protein Determination Measured via BCA

The protein content was determined in all the samples using spectrophotometry in a solution with bicinchoninic acid assay (BCA) following the procedure described by Gringer et al. (2015) [1].

### 3.5. Element Determination

Determination of elements in cod process waters before and after filtration and flocculation was performed. In the current study, the nutritional elements zinc (Zn), copper (Cu), iron (Fe), manganese (Mn), calcium (Ca), potassium (K), phosphorus (P), magnesium (Mg), sodium (Na) and the potentially toxic element cadmium (Cd) were determined using inductively coupled plasma mass spectrometry (ICP-MS) (iCAPq, Thermo-Fischer, Bremen, Germany) following digestion of the samples with concentrated nitric acid (SPS Science, France) using a microwave oven (Multiwave 7000, Anton Paar, Graz, Austria) [36]. Quantification was performed via external calibration with standard solutions prepared from certified stock solutions (SPS Science) and using rhodium as internal standard (SPS Science). A certified reference material, DORM-4 (fish muscle) (NRCC, Ottawa, ON, Canada), was analyzed (*n* = 4) together with the samples, and the obtained values were in good agreement with the certified reference values.

### 3.6. Polypeptide Profiling Using SDS-PAGE

The molecular weight distribution (MWD) of the untreated and treated samples was determined via gel electrophoresis using10% Bis-Tris 12 well gels (NuPAGE^TM^, Invitrogen, Thermo Fisher, Whaltham, MA, USA).

Sample solutions of 1 mg/mL of protein were prepared. A solution with 250 µL Laemlli buffer and 25 µL of DTT per sample was prepared. Then, 250 µL of the previous Laemlli-DTT solution was added to 250 µL of each sample solution in Eppendorf tubes. These were heated in a water bath (100 °C, 3 min), then centrifuged for 3 min at 12,000 rpm (Biofuge pico, Heraeus, Osterode, Germany). The running buffer solution was prepared by mixing 40 mL of running buffer with distilled water to a total volume of 800 mL and put in the outer space of the cell chamber (XCell *Sure*Lock^TM^ Mini Cell, Thermo Fisher, Whaltham, MA, USA) that had the gel in its cassette inserted. The first and last wells of the gel were loaded with standard (5 µL), while 10 µL of each sample were loaded to the rest of the wells in the gel. The gels ran with a voltage of 200 V in the electrophoresis equipment for approximately 45 min. Afterwards, the gel was rinsed with distilled water (5 min × 3). Then, the stain reagent was added and left for 1 h. After this time, the stain was removed and distilled water was added and left for 24 h to remove excess of stain. Finally, the bands from the samples were observed and compared with the standard.

### 3.7. Data Treatment

After obtaining the protein concentration (mg/mL) and phosphorus concentration (mg/kg), Equation (1) was applied to obtain the protein content (%) and phosphorus content (%) in the supernatants after the flocculation processes, and the recovered protein (%) and recovered phosphorus (%) in the phases after the treatments with membrane technology.

(1) $$Nutrient\;content\left(\%\right)=\frac{Nutrient\;concentration\;of\;separated\;sample}{Nutrient\;concentration\;in\;the\;untreated\;sample}\times 100$$

Then, to calculate the sedimented protein (%) and sedimented phosphorus (%) after the flocculation treatments, Equation (2) was used.

(2) $$Sedimented\;nutrient\left(\%\right)=100-Nutrient\;content\;(\%)$$

For the mass balance in the membrane technology treatment, the protein contents (mg) of the separated and treated samples were calculated. For this, Equation (3) was used.

(3) $$Protein\;content\left(mg\right)=Protein\;conc.of\;untreatead\;or\;separated\;samples\left[\frac{mg}{mL}\right]\times Sample\;volume\;(mL)$$

The phosphorus content (mg) was calculated from the phosphorus concentration using Equation (4) to obtain the phosphorus mass balance.

(4) $$Phosphorus\;content\left(mg\right)=Phosph.\;conc.\;of\;untreated\;or\;separated\;sample\left[\frac{mg}{kg}\right]\times (Sample\;mass\;(g)/1000)$$

Equations (1), (2) and (4) were applied to calculate sedimented minerals (%) after the flocculation treatments and recovered minerals (%) in the phases after the filtrations.

Statistical analysis was performed when at least duplicates were available for the data set to be analyzed using Microsoft Excel and Minitab Statistical Software (Version 2022, Coventry, UK). The main statistical tests used were one-way and multifactorial ANOVA, being a value of *p* = 0.05 or less considered to have a significant difference between the variables compared. Tukey analyses were also used for multiple comparison of means. Linear regression analyses were applied to find the relationship of sedimentation or recovery between phosphorus and protein after the treatments. Specific samples were selected for the analysis of phosphorus content, and single determination was performed per sample. Therefore, statistical analysis for these results could not be conducted.

## 4. Conclusions

Process waters LS and HS followed different patterns with respect to protein and phosphate recovery when different flocculant concentrations and maturation times were used. Proteins in LS could be most efficiently recovered by using Levasil RD442 0.25% and no maturation period (51.4%), while phosphate was most efficiently recovered when using Levasil RD442 1.23% and a maturation period of 1 h (34.7%). For HS, most of its protein was recovered using Levasil RD442 1.23% and a maturation period of 1 h (51.8%), while phosphate was recovered the most using Levasil 1.23% and no maturation period (47.1%). These differences were possibly due to the salt content of each process water and the interactions through intermolecular forces that exist with the amounts of flocculant and the salts in each process water. Chitosan lactate acts as a flocculant with high charge density on solutions with a pH below 5, and the pH of the process waters that were used was above 6, not allowing electrostatic interactions between the flocculant and the proteins. Chitosan lactate requires the step of pH adjustment included in the coagulation/flocculation methodology, which can be more difficult and expensive to perform. A flocculation treatment that can be applied to both process waters is Levasil RD442 1.23% and no maturation period, since this would recover high percentages of protein (37% in LS and 43% in HS) and phosphate (31% in LS and 47% in HS).

Ultrafiltration was more effective on HS since it recovered a high percentage of both protein (57%) and phosphate (46%) in the retentate phase, which is the phase with less volume and one that can be later processed for further uses. This is not the case for LS, since a large percentage of protein (40%) stayed in the filtration system and required rinsing steps to recover it, while the phosphate went mainly to the permeate phase (82%), which has a big volume, and it is technically not a separation. These differences can be attributed to the densities and particles present in each process water. Microfiltration was not successful due to the large pore size of the membrane, not allowing a separation of the nutrients.

Regarding other minerals, Na could not be concentrated with the use of an ultrafiltration membrane. The treatment with Levasil RD442 1.23% without a maturation period showed the highest recovery (8.8%) of Na for LS, while for HS, the treatment with Levasil RD442 1.23% with a maturation period of 1 h recovered most of this mineral (5.8%). Cu and Zn were highly concentrated in the retentate phase after the ultrafiltration treatment.

## Figures and Tables

**Table 1:** Salt content and protein content of the process waters LS and HS from the production of salted cod at Jeka Fish. These values were determined in previous batches and were only used as a reference to select high protein content process waters. Protein measured using Kjeldahl method.

Sample	Protein [%]	Salt [%]
LS	0.91	6.95–13.44
HS	0.85–1.06	22.75–25.63

**Table 2:** Protein content of the process waters LS and HS from the production of salted cod at Jeka Fish. These values were determined in the batches that were used for the separation processes evaluated in this study.

Sample	Date of Collection	Protein [%]
LS	9 March 2022	0.24 ± 0.01
	28 March 2022	0.41 ± 0.02
	25 April 2022	0.10 ± 0.02
HS	21 February 2022	0.28 ± 0.02
	28 March 2022	0.35 ± 0.01
	25 April 2022	0.39 ± 0.02

## Data Availability

Data are contained within the article.

## Supplementary Materials

The following supporting information can be downloaded at: https://www.mdpi.com/article/10.3390/md21110558/s1, Table S1. Determination of minerals and their recovery after treatments of flocculation and use of membrane technology to selected samples of process waters LS and HS from different dates.
