# Supplementary material for: Recovery of Nutrients from Cod Processing Waters
Source: Mar Drugs. 2023 Oct 26;21(11):558. doi: 10.3390/md21110558 (PMC10672049; doi:10.3390/md21110558)
Supplement: Supplementary file 1 [file marinedrugs-21-00558-s001.zip › marinedrugs-2509045-supplementary.pdf]

Supplementary Materials:

Table S1. Determination of minerals and their recovery after treatments of flocculation and use of membrane technology to selected samples of process waters LS and HS from different dates. LOD = Level of Detection, LOQ = Level of Quantification.

		Element	Ca	Ca	Mn	Mn	Fe	Fe	Cu	Cu	Zn	Zn	Cd
		Isotope used for determination (amu)	44	Sedimented or recovered	55	Sedimented or recovered	57	Sedimented or recovered	63	Sedimented or recovered	66	Sedimented or recovered	111
		unit	g/kg	%	mg/kg	%	mg/kg	%	mg/kg	%	mg/kg	%	mg/kg
Sample	Collected in	Description											
LS	9-Mar	No treatment	0.103		0.044		0.277		0.006		0.255		<LOQ
LS	9-Mar	Levasil 1.23% and 0 hour	0.069	32.6	0.006	85.1	0.085	69.2	0.006	-0.2	0.080	68.7	<LOQ
HS	21-Feb	No treatment	0.179		0.124		0.447		0.011		0.796		<LOQ
HS	21-Feb	Levasil 1.23% and 0 hour	0.087	51.4	0.010	92.1	0.147	67.0	0.007	32.5	0.060	92.5	<LOQ
LS	25-Apr	No treatment	0.149		0.050		0.370		0.069		0.119		<LOQ
LS	25-Apr	Levasil 0.15% and 1 hour	0.115	22.9	0.017	65.6	0.290	21.5	<LOD		0.011	90.6	<LOQ
LS	25-Apr	Levasil 0.25% and 1 hour	0.114	23.6	0.016	67.6	0.288	22.2	0.050	27.7	0.066	44.9	<LOQ
LS	25-Apr	Levasil 1.23% and 1 hour	0.105	29.4	0.010	80.6	0.135	63.6	<LOD		<LOD		<LOQ
LS	25-Apr	Levasil 0.15% and 3 hours	0.112	24.9	0.008	83.3	0.248	33.0	<LOD		<LOD		<LOQ
LS	25-Apr	Levasil 0.25% and 3 hours	0.102	32.0	0.010	80.5	0.205	44.6	<LOD		0.006	95.0	<LOQ
LS	25-Apr	Levasil 1.23% and 3 hours	0.099	33.8	0.013	73.2	0.166	55.1	0.003	95.2	0.021	82.5	<LOQ
HS	25-Apr	No treatment	0.083		0.017		0.415		0.011		0.170		<LOQ
HS	25-Apr	Levasil 0.15% and 1 hour	0.061	26.1	0.009	45.1	0.395	4.8	0.003	67.6	0.158	7.2	<LOQ
HS	25-Apr	Levasil 0.25% and 1 hour	0.060	27.8	0.009	48.8	0.338	18.6	0.003	70.2	0.094	44.9	<LOQ
HS	25-Apr	Levasil 1.23% and 1 hour	0.053	36.2	0.010	41.9	0.206	50.5	0.009	16.1	0.005	97.0	<LOQ
HS	25-Apr	Levasil 0.15% and 3 hours	0.059	28.4	0.009	47.4	0.360	13.2	0.004	61.6	0.113	33.6	<LOQ
HS	25-Apr	Levasil 0.25% and 3 hours	0.058	30.3	0.010	40.3	0.369	11.2	0.060	-458.2	0.112	34.1	<LOQ
HS	25-Apr	Levasil 1.23% and 3 hours	0.058	29.5	0.009	44.3	0.240	42.2	0.005	53.5	0.019	88.7	<LOQ
LS	28-Mar	No treatment	0.140		0.042		0.398		0.024		0.645		<LOQ
LS	28-Mar	Permeate A - Ultrafiltration	0.145	103.7	0.021	48.5	0.304	76.4	0.521	2132.0	2.378	368.7	<LOQ
LS	28-Mar	Permeate B - Ultrafiltration	0.147	104.9	0.022	51.1	0.349	87.8	0.440	1801.8	1.826	283.1	<LOQ
LS	28-Mar	Permeate C - Ultrafiltration	0.144	103.1	0.026	60.2	0.415	104.3	0.415	1698.2	2.094	324.7	<LOQ
LS	28-Mar	Retentate - Ultrafiltration	0.340	243.8	0.129	304.2	1.382	347.2	14.171	57996.0	12.713	1971.2	<LOQ
HS	28-Mar	No treatment	0.087		0.020		0.617		0.012		0.228		<LOQ
HS	28-Mar	Permeate A - Ultrafiltration	0.055	62.8	0.008	41.8	0.336	54.6	1.251	10094.9	1.524	667.3	<LOQ
HS	28-Mar	Permeate B - Ultrafiltration	0.053	60.8	0.006	29.8	0.414	67.1	1.179	9515.0	1.587	695.3	<LOQ
HS	28-Mar	Permeate C - Ultrafiltration	0.048	55.4	0.006	29.2	0.417	67.6	0.934	7533.4	1.616	707.9	<LOQ
HS	28-Mar	Retentate - Ultrafiltration	0.134	153.3	0.029	145.9	0.862	139.8	3.138	25318.3	2.372	1038.8	<LOQ

		Element	Na	Na	Mg	Mg	P	P	K	K
		Isotope used for determination (amu)	23	Sedimented or recovered	24	Sedimented or recovered	31	Sedimented or recovered	39	Sedimented or recovered
		unit	g/kg	%	mg/kg	%	g/kg	%	g/kg	%
Sample	Collected in	Description								
LS	9-Mar	No treatment	50.6		74.0		1.16		0.485	
LS	9-Mar	Levasil 1.23% and 0 hour	46.1	8.8	4.6	93.7	0.80	31.4	0.410	15.4
HS	21-Feb	No treatment	39.9		398.4		2.76		0.752	
HS	21-Feb	Levasil 1.23% and 0 hour	38.6	3.1	5.5	98.6	1.46	47.2	0.767	-2.0
LS	25-Apr	No treatment	53.4		167.8		1.76		0.192	
LS	25-Apr	Levasil 0.15% and 1 hour	57.0	-6.6	34.1	79.7	1.43	18.8	0.196	-1.9
LS	25-Apr	Levasil 0.25% and 1 hour	55.3	-3.5	33.5	80.0	1.40	20.5	0.208	-8.0
LS	25-Apr	Levasil 1.23% and 1 hour	50.9	4.7	13.0	92.2	1.15	34.9	0.182	5.3
LS	25-Apr	Levasil 0.15% and 3 hours	55.7	-4.3	11.9	92.9	1.32	24.8	0.224	-16.4
LS	25-Apr	Levasil 0.25% and 3 hours	55.0	-3.0	14.7	91.2	1.35	23.5	0.201	-4.6
LS	25-Apr	Levasil 1.23% and 3 hours	51.0	4.5	22.7	86.4	1.20	32.0	0.194	-1.2
HS	25-Apr	No treatment	103.1		15.1		1.95		1.005	
HS	25-Apr	Levasil 0.15% and 1 hour	99.2	3.8	2.7	82.3	1.83	6.2	0.936	6.9
HS	25-Apr	Levasil 0.25% and 1 hour	99.1	3.9	3.2	78.6	1.89	3.1	0.950	5.5
HS	25-Apr	Levasil 1.23% and 1 hour	97.1	5.8	2.0	86.5	1.73	11.0	0.892	11.2
HS	25-Apr	Levasil 0.15% and 3 hours	98.9	4.1	3.0	80.3	1.85	4.9	0.965	4.0
HS	25-Apr	Levasil 0.25% and 3 hours	97.7	5.3	3.2	78.6	1.84	5.4	0.973	3.2
HS	25-Apr	Levasil 1.23% and 3 hours	99.8	3.2	2.5	83.6	1.81	7.1	0.917	8.7
LS	28-Mar	No treatment	29.9		40.7		2.67		1.025	
LS	28-Mar	Permeate A - Ultrafiltration	24.0	80.4	26.4	64.9	1.87	70.0	0.809	79.0
LS	28-Mar	Permeate B - Ultrafiltration	28.5	95.6	19.2	47.1	2.34	87.5	0.954	93.1
LS	28-Mar	Permeate C - Ultrafiltration	28.3	94.7	20.2	49.6	2.60	97.4	0.975	95.1
LS	28-Mar	Retentate - Ultrafiltration	31.3	104.9	158.5	389.5	4.39	164.4	1.023	99.8
HS	28-Mar	No treatment	101.4		19.6		1.89		0.870	
HS	28-Mar	Permeate A - Ultrafiltration	86.8	85.6	13.5	69.1	1.22	64.6	0.707	81.4
HS	28-Mar	Permeate B - Ultrafiltration	101.6	100.3	4.7	23.8	1.60	84.7	0.871	100.2
HS	28-Mar	Permeate C - Ultrafiltration	100.3	99.0	5.1	26.1	1.55	82.1	0.861	99.0
HS	28-Mar	Retentate - Ultrafiltration	101.4	100.1	28.1	143.6	1.91	101.3	0.845	97.1
